# Bilirubin Decreases Macrophage Cholesterol Efflux and ATP‐Binding Cassette Transporter A1 Protein Expression

**DOI:** 10.1161/JAHA.117.005520

**Published:** 2017-04-28

**Authors:** Dongdong Wang, Anela Tosevska, Elke H. Heiß, Angela Ladurner, Christine Mölzer, Marlies Wallner, Andrew Bulmer, Karl‐Heinz Wagner, Verena M. Dirsch, Atanas G. Atanasov

**Affiliations:** ^1^ Department of Pharmacognosy University of Vienna Austria; ^2^ Institute of Genetics and Animal Breeding of the Polish Academy of Sciences Jastrzebiec Poland; ^3^ Research Platform Active Ageing University of Vienna Austria; ^4^ Department of Nutritional Sciences University of Vienna Austria; ^5^ Department of Molecular, Cell and Developmental Biology UCLA Los Angeles CA; ^6^ School of Medicine, Medical Sciences and Nutrition Institute of Medical Sciences University of Aberdeen United Kingdom; ^7^ Institute of Dietetics and Nutrition University of Applied Sciences FH JOANNEUM Graz Austria; ^8^ School of Medical Science and Menzies Health Institute Queensland Gold Coast Australia

**Keywords:** ATP‐binding cassette transporter, bilirubin, cardiovascular disease, cholesterol, cholesterol homeostasis, Atherosclerosis, Cardiovascular Disease, Lipids and Cholesterol

## Abstract

**Background:**

Mild but chronically elevated circulating unconjugated bilirubin is associated with reduced total and low‐density lipoprotein cholesterol concentration, which is associated with reduced cardiovascular disease risk. We aimed to investigate whether unconjugated bilirubin influences macrophage cholesterol efflux, as a potential mechanism for the altered circulating lipoprotein concentrations observed in hyperbilirubinemic individuals.

**Methods and Results:**

Cholesterol efflux from THP‐1 macrophages was assessed using plasma obtained from normo‐ and hyperbilirubinemic (Gilbert syndrome) humans (n=60 per group) or (heterozygote/homozygote Gunn) rats (n=20 per group) as an acceptor. Hyperbilirubinemic plasma from patients with Gilbert syndrome and Gunn rats induced significantly reduced cholesterol efflux compared with normobilirubinemic plasma. Unconjugated bilirubin (3–17.1 μmol/L) exogenously added to plasma‐ or apolipoprotein A1–supplemented media also decreased macrophage cholesterol efflux in a concentration‐ and time‐dependent manner. We also showed reduced protein expression of the ATP‐binding cassette transporter A1 (ABCA1), a transmembrane cholesterol transporter involved in apolipoprotein A1–mediated cholesterol efflux, in THP‐1 macrophages treated with unconjugated bilirubin and in peripheral blood mononuclear cells obtained from hyperbilirubinemic individuals. Furthermore, we demonstrated that bilirubin accelerates the degradation rate of the ABCA1 protein in THP‐1 macrophages.

**Conclusions:**

Cholesterol efflux from THP‐1 macrophages is decreased in the presence of plasma obtained from humans and rats with mild hyperbilirubinemia. A direct effect of unconjugated bilirubin on cholesterol efflux was demonstrated and is associated with decreased ABCA1 protein expression. These data improve our knowledge concerning bilirubin's impact on cholesterol transport and represent an important advancement in our understanding of bilirubin's role in cardiovascular disease.

## Introduction

For many years, bilirubin, the end product of heme catabolism, was regarded as a harmful waste product^1^ and was assumed to possess little or no biological function.^2^ More recent evidence, however, suggests an inverse relationship between circulating unconjugated bilirubin (UCB) and the risk of cardiovascular disease (CVD).^1^ In 1994, Schwertner et al^3^ first reported that serum bilirubin concentrations in the upper physiological range (10–17.1 μmol/L)^3^ were associated with reduced prevalence of coronary artery disease. Subsequently, a number of retrospective and prospective studies indicated that high‐normal (10–17.1 μmol/L)^3^ serum bilirubin is associated with decreased risk of CVD.^4^, ^5^, ^6^, ^7^, ^8^, ^9^, ^10^, ^11^

These reports, however, do not conclusively implicate a cardiovascular protective effect of mildly elevated UCB concentrations and demonstrate associations between cardiovascular risk factors in cohorts of subjects with Gilbert syndrome (GS), in the range of 17.1 to 90 μmol/L.^12^ GS is a genetic disorder resulting in a benign, mildly elevated UCB serum concentration with a prevalence of 5% to 10% in the white population.^2^, ^12^, ^13^ The main cause of GS is a hereditary mutation in the promoter of the uridine diphosphate‐glucuronosyltransferase (*UGT*) gene, which leads to reduced hepatic UGT 1 family, polypeptide A1 (UGT1A1) expression.^14^ UGT1A1 is primarily responsible for conjugating bilirubin with glucuronic acid, allowing its excretion via the bile from the circulation.^15^ In addition to bilirubin, UGT1A1 is involved in the glucuronidation of 17β‐estradiol, 17α‐ethinylestradiol, and some xenobiotics.^16^ Nevertheless, no evidence currently exists to demonstrate that this UGT1A1 polymorphism affects hormone levels in postmenopausal women.^17^

Interestingly, patients with GS have significantly reduced incidence of ischemic heart disease^18^ and significant delay in developing clinically relevant manifestations of CVD^19^ compared with the general population. Furthermore, homozygous carriers of the UGT1A1*28 allele, characterized by the presence of an additional TA repeat in the TATA sequence of the *UGT1A1* promoter—(TA)_7_TAA instead of (TA)_6_TAA)^8^—with mildly elevated serum UCB concentrations, also demonstrate a reduced risk of CVD.^8^

Elevated serum UCB concentrations are also reported in Gunn rats.^18^ Gunn rats inherit a single point mutation in the coding region of the *UGT1A1* gene that truncates and inactivates UGT1A1, leading to complete absence of bilirubin glucuronidation capacity.^2^, ^20^ UCB serum concentrations of these animals range between 50 and 200 μmol/L.^21^ In line with observations in human GS, hyperbilirubinemia beneficially modulated myocardial function and aortic ejection and imparted ischemic stress resistance in Gunn rats.^22^

Although a body of evidence indicates that upper normal (10–17.1 μmol/L)^3^ or mildly elevated (17.1–90 μmol/L)^12^ plasma bilirubin levels are associated with a reduced risk of CVD, conflicting reports show varying binomial relationships,^23^, ^24^, ^25^, ^26^, ^27^, ^28^ with a recent report suggesting that humans with higher bilirubin levels (12–86 μmol/L)^29^ have a risk similar to that of persons with the lowest bilirubin levels (<7 μmol/L).^29^ The studies show a U‐shaped relationship between circulating bilirubin concentrations and risk of ischemic heart disease, suggesting that both lower and higher concentrations of serum bilirubin are associated with an increased risk of CVD.^29^, ^30^ Similarly, a U‐shaped association of total bilirubin levels with all‐cause mortality was also demonstrated.^31^, ^32^ So far, the molecular determinants of this complex bioactivity pattern remain elusive; however, they are most likely explained by the inclusion of patients with underlying hepatic damage, which confounds protective associations.^32^

At present, several plausible mechanisms have been suggested to play a potential role in the antiatherogenic and cardioprotective activity of bilirubin.^1^ The most commonly proposed mechanism is bilirubin's antioxidant capacity that prevents lipid and lipoprotein peroxidation, a process involved in the pathophysiology of atherosclerosis.^1^, ^33^, ^34^, ^35^, ^36^ Furthermore, bilirubin inhibits vascular inflammation^4^, ^34^, ^35^ and immune cell proliferation.^1^ Moreover, recent studies suggest that bilirubin inhibits vascular smooth muscle cell proliferation and migration,^10^, ^37^, ^38^ as well as endothelial dysfunction,^39^, ^40^ which are important steps in the atherosclerotic process. In addition, patients with GS are reported to have improved resistance to serum oxidation,^32^, ^34^ altered inflammatory responses,^35^, ^36^ and modified lipid status and metabolism,^2^, ^41^, ^42^, ^43^ all of which likely contribute to cardiovascular protection in GS. Similar protective effects were also demonstrated in the Gunn rats.^2^, ^10^, ^44^, ^45^

Although bilirubin appears to affect multiple steps in the atherosclerotic process, it remains to be established whether variations of UCB plasma concentrations influence macrophage cholesterol efflux, which is a promising target for the prevention and treatment of CVD.^41^, ^42^ Clinical reports indicate that macrophage cholesterol efflux is significantly and inversely associated with CVD, independent of high‐density lipoprotein cholesterol (HDL‐C) concentrations,^41^, ^42^, ^43^ suggesting that the cholesterol efflux capacity may be a novel predictive biomarker for the incidence of cardiovascular events.^46^ A well‐established pathway of macrophage cholesterol efflux involves apolipoprotein A1 (apo A1; the major protein in HDL) as an acceptor and membrane‐associated transporter ATP‐binding cassette transporter A1 (ABCA1).^47^, ^48^ ABCA1 promotes cholesterol efflux from macrophages to lipid‐poor apo A1 (often referred to as *pre‐β HDL*).^49^ ABCA1 is confirmed as a major mediator of cholesterol efflux to HDL.^50^, ^51^

In the current study, we compared cholesterol efflux mediated by plasma from patients with GS or Gunn rats with that from normobilirubinemic humans and rats, respectively. Moreover, we evaluated the influence of UCB on macrophage cholesterol efflux and ABCA1 protein expression and degradation.

## Materials and Methods

### Human Participants

Sixty patients with GS and 60 age‐ and sex‐matched healthy controls were recruited according the inclusion and exclusion criteria described by Mölzer et al^52^ and Tosevska et al.^53^ Methods used for plasma sample preparation, peripheral blood mononuclear cell (PBMC) isolation, age distribution, UCB levels, and blood biochemistry were described by Mölzer et al.^52^ The study was approved by the ethics committee of the Medical University of Vienna and the General Hospital of Vienna (no. 1164/2014) and conducted in accordance with the guidelines approved by the Declaration of Helsinki. All participants provided written informed consent.

### Animals

Plasma samples from 20 hyperbilirubinemic Gunn rats (homozygous for a mutation in UGT1A1, 10 male and 10 female) and 20 respective controls (10 male and 10 female), normobilirubinemic Wistar rats (heterozygous for a mutation in *UGT1A1*) were collected and stored at −80°C, as described previously.^54^ The Gunn rat is a spontaneous mutant strain bred from Wistar rats,^50^ and both Gunn and Wistar rats had the same genetic background apart from homo/heterozygosity for *UGT1A1*.^50^ The study was approved by the committee of animal experiments of the Austrian Federal Ministry of Science and Research (BMF‐66.006/0008‐II/3b/2011).^54^

### Cholesterol Efflux

Quantification of cholesterol efflux was performed, as described previously.^51^, ^55^, ^56^ THP‐1 cells (ATCC) were maintained in RPMI‐1640 medium (Lonza) supplemented with 10% fetal bovine serum (Gibco), 100 U/mL penicillin and 100 μg/mL streptomycin, and 2 mmol/L l‐glutamine (Invitrogen) at 37°C and 5% CO_2_. THP‐1 cells were seeded at 0.2×10^6^ cells per well in 24‐well plates and differentiated into macrophages for 72 hours with 200 nmol/L phorbol‐12‐myristate‐13‐acetate (catalog no. P1585; Sigma‐Aldrich). Cells were washed twice with PBS, then labeled by incubation in RPMI‐1640 medium supplemented with 0.1% (wt/vol) fatty acid‐free bovine serum albumin and [^3^H]‐cholesterol (0.5 μCi/mL, catalog no. NET139001MC; PerkinElmer) for 24 hours.

To evaluate cholesterol efflux mediated by plasma from human participants and rats, labeled cells were washed and then incubated with fresh serum‐free medium containing plasma (3%, vol/vol) from human participants or rats for 4 hours. To evaluate an effect of added bilirubin on cholesterol efflux to normobilirubinemic plasma, labeled cells were washed and then incubated with fresh serum‐free medium containing plasma (3%, vol/vol) in the absence or presence of UCB IXα (17.1 μmol/L) for 4 hours. For the concentration‐ and time‐dependent experiments, labeled cells were washed with PBS and treated with different concentrations of UCB (3, 10, or 17.1 μmol/L) or solvent vehicle (0.1% dimethyl sulfoxide) for different time periods (4, 8, 16, or 24 hours). Cells were washed again with PBS and then incubated with fresh serum‐free medium containing human plasma (3%, vol/vol) or apo A1 (10 μg/mL, catalog no. SRP4693; Sigma‐Aldrich) for 4 hours. To differentiate whether UCB inhibits cholesterol efflux by interacting with THP‐1 macrophages or with cholesterol acceptors, labeled cells were washed and then treated with UCB (17.1 μmol/L) or solvent vehicle (0.1% dimethyl sulfoxide) for 4 hours. Cells were washed again with PBS and then incubated with fresh serum‐free medium containing human plasma (3%, vol/vol) or apo A1 (10 μg/mL; Sigma‐Aldrich) in the absence or presence of UCB (17.1 μmol/L) for 4 hours. Effluxed (medium supernatant) and intracellular (cell lysate) [^3^H]‐cholesterol were counted by liquid scintillation. Cholesterol efflux (percentage of total cholesterol) was determined by the ratio of radio‐labeled cholesterol in the medium to that of both medium and cells.

### Cell Viability

Cell viability was assessed by the resazurin conversion assay.^56^, ^57^ For this assay, THP‐1 cells were seeded at 0.4×10^5^ cells per well in 96‐well plates and differentiated, then loaded with unlabeled cholesterol and treated with UCB, as described in the previous section. Afterward, cells were incubated with PBS containing 10 μg/mL resazurin for 4 hours. The relative cell viability was quantified from the increased fluorescent signal by the conversion product resorufin by measuring the fluorescence at 590‐nm emission/535‐nm excitation with a Tecan GENiosPro plate reader.

### Trypan Blue Exclusion Assay

THP‐1 cells were seeded at 0.8×10^6^ cells per well in 6‐well plates and differentiated. Then cells were loaded with unlabeled cholesterol, as described previously. Cells were treated with increasing concentrations of bilirubin (1–17.1 μmol/L) for 4, 8, 16, and 24 hours. Cells were detached using Accutase solution (catalog no. A6964, Sigma) (1 mL/well). Cellular integrity was assessed by the Trypan blue exclusion test using a Vi‐CELL cell counter (Beckman Coulter GMBH).

### Western Blot Analysis

Protein extraction and western blot analysis were performed, as described previously.^57^, ^58^, ^59^ THP‐1 cells were seeded at 0.8×10^6^ cells per well in 6‐well plates and differentiated. Then cells were loaded with unlabeled cholesterol, as described previously. Cells were washed again with PBS and then treated with UCB (17.1 μmol/L) for 4, 8, 16, and 24 hours. To evaluate the effect of bilirubin on upregulated ABCA1 protein expression, THP‐1 macrophages were treated with an liver X receptor (LXR) agonist (TO901317, 5 μmol/L) or solvent vehicle (0.1% dimethyl sulfoxide) for 24 hours prior to exposure to bilirubin (17.1 μmol/L) or solvent vehicle (0.1% dimethyl sulfoxide) for another 16 hours. In addition, PBMCs were extracted from whole blood of human participants, as described by Mölzer et al.^52^ Cells (THP‐1–derived macrophages or PBMCs) were lysed in ice‐cold NP‐40 lysis buffer (150 mmol/L NaCl; 50 mmol/L HEPES (pH 7.4); 1% NP‐40; 1% PMSF; 0.5% Na_3_VO_4_; 0.5% NaF) containing Complete protease inhibitor (Roche) for 30 minutes before centrifugation to remove cellular debris. Total cell protein was measured according to the Bradford method.

Protein extraction from liver tissue was performed, as described previously.^60^ Overall, 50 mg of liver tissue from Gunn rats and Wistar rats were homogenized in ice‐cold NP‐40 lysis buffer containing Complete protease inhibitor (Roche) using sonication (Bandelin Electronic, Sonopuls HD 2070). The Sonopuls HD 2070 was set as follows: power: H position (high); sonication cycle: 15 seconds on/15 seconds off; total sonication time: 5 to 15 cycles; temperature: 4°C. After sonication, the lysate was centrifuged at 16 060*g* for 20 minutes. The supernatant was collected, and protein concentration was determined by Bradford assay.

Samples were separated by SDS‐PAGE and transferred to a polyvinylidene fluoride membrane. After blocking for 1 hour with 5% nonfat milk in TBS‐Tween, membranes were incubated with the following primary antibodies at 4°C overnight: ABCA1 (catalog no. NB400‐105, 1:500; Novus), β‐actin (catalog no. 8691002, 1:5000; MP Biomedicals). Goat antimouse (catalog no. 12‐349, 1:5000; MP Biomedicals) or antirabbit (catalog no. 7074S 1:500; Cell Signaling) secondary antibodies were utilized, according to the manufacturer's instructions. Protein bands were visualized with the Fuji LAS 3000 CCD camera (Fujifilm) and quantified with AIDA software (Raytest GmbH).

### RNA Extraction and Quantitative Reverse Transcription Polymerase Chain Reaction

THP‐1 cells were differentiated, loaded, and treated as described earlier. Total RNA was extracted from differentiated THP‐1 macrophages using the peqGOLD Total RNA Kit (PeqLab), according to the manufacturer's instructions.^56^ The quantitation of RNA was performed with NanoDrop 2000c (peqlab; Thermo Scientific). A ratio of the absorbance at 260 and 280 nm (A_260/280_) close to 2.0 was considered to indicate sufficient RNA quality. cDNA was synthesized with 1 μg total RNA based on the protocol from the High Capacity cDNA Reverse Transcription Kit (Applied Biosystems) with RNase Inhibitor (Applied Biosystems). Quantitative reverse transcription polymerase chain reaction was conducted using the LightCycler 480 SYBR Green I Master Kit (Roche) with 40 ng cDNA for each sample. A LightCycler 480 system of Roche was used for detection of the amplification cycles. Primers used for quantitative reverse transcription polymerase chain reaction were specific for ABCA1 (HS_ABCA1_1_SG QuantiTect primer assay, catalog no. QT00064869; Qiagen), ATP‐binding cassette transporter G1 (ABCG1; Hs_ABCG1_1_SG QuantiTect Primer Assay, catalog no. QT00021035; Qiagen), and 18S (Hs_RRN18S_1_SG QuantiTect Primer assay, catalog no. QT00199367; Qiagen). Relative quantification of *ABCA1* and *ABCG1* gene expression was performed with the ΔC_T_ method, using 18S as endogenous control.

### Quantification of ABCA1 Degradation Rate

THP‐1 cells were seeded at 0.8×10^6^ cells per well in 6‐well plates and differentiated, as described earlier.^61^ Cells were loaded with unlabeled cholesterol, as described. Cells were treated with bilirubin (final concentration 17.1 μmol/L) for 8 hours. Control cells were treated with solvent vehicle (0.1% dimethyl sulfoxide) for 8 hours. Cells were lysed at different time points after treatment with the protein synthesis inhibitor cycloheximide (140 μmol/L; 0, 10, 20, 40, 60, and 80 minutes). The protein levels of ABCA1 were detected by western blot analysis.

### Statistical Analyses

For determination of differences between 2 groups, a 2‐tailed unpaired Student *t* test or paired *t* test was applied after data were tested for normality. For multiple comparisons, data were analyzed by 1‐way ANOVA followed by Bonferroni post hoc test to compare means between groups. *P*<0.05 was considered statistically significant. Forward stepwise regression analysis was utilized to predict the effect of multiple independent variables on cholesterol efflux capacity. GraphPad Prism (version 4.03; GraphPad Software ) was used for statistical analyses and figure generation.

## Results

### Plasma of Gilbert Syndrome Patients and Gunn Rats With Hyperbilirubinemia Mediates Decreased Cholesterol Efflux

**Figure 1:**
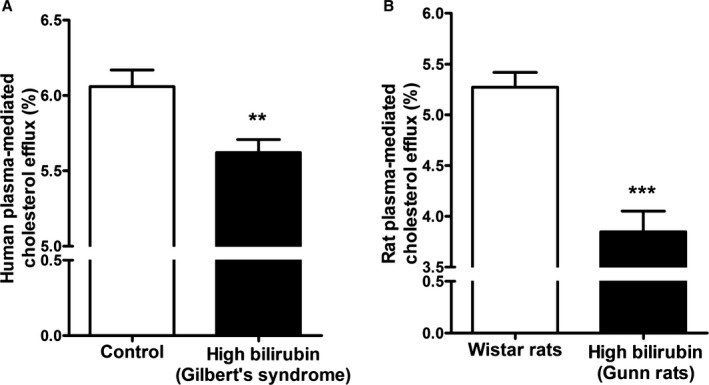
Plasma cholesterol efflux capacity is impaired in the presence of human (A) and rat (B) plasma with high unconjugated bilirubin. THP‐1 cells were differentiated for 72 hours with 200 nmol/L phorbol‐12‐myristate‐13‐acetate and then loaded with unlabeled cholesterol and radioactive cholesterol tracer ([^3^H]‐cholesterol) for another 24 hours. Cells were then incubated with fresh serum‐free medium containing (A) human plasma (3%, vol/vol) from Gilbert syndrome patients or control participants (n=60 per group), or (B) rat plasma (3%, vol/vol) from Gunn or Wistar rats (n=20 per group) for 4 hours, and cholesterol efflux was determined. Values are mean±SEM. All cholesterol efflux experiments were performed in triplicate for each sample. **P<, 0.01,***P<0.001 vs the control participants or Wistar rats group (determined by paired *t* test).

To assess differences in cholesterol efflux mediated by normo‐ and hyperbilirubinemic human plasma, we tested plasma samples of 60 GS patients and 60 age‐ and sex‐matched controls. The cholesterol efflux mediated by plasma from participants with GS was 7.2% lower than that from control participants (*P*<0.0001) (Figure 1A). Interestingly, plasma from hyperbilirubinemic Gunn rats also demonstrated a similar result: The cholesterol efflux mediated by Gunn rat plasma was 27.0% lower than normobilirubinemic control plasma from Wistar rats (*P*<0.0001) (Figure 1B).

Quantitative characteristics of the plasma used in this study were recently described.^52^ HDL‐C (*P*=0.726), apo A1 (*P*=0.638), and apo B (*P*=0.194) did not differ significantly between GS and control participants.^52^ In addition, there was no difference in low‐density lipoprotein cholesterol (LDL‐C) or oxidized LDL/LDL ratio in this study population (K.‐H. Wagner, unpublished data, 2015). There was a significant difference (*P*<0.001) in the concentration of UCB between GS patients (33±10 μmol/L) and control participants (9±3 μmol/L).^52^ Data of UCB concentrations, blood biochemistry, and body mass in Gunn and Wistar rats were also published previously.^54^ HDL‐C levels in Gunn rats were significantly lower than those in Wistar rats (*P*<0.001); however, no data were available on apo A1 or apo B levels.^54^ UCB concentrations were also significantly elevated in Gunn rats (106±26 μmol/L) compared with Wistar control (0.57±0.19 μmol/L).^54^

### Multiple Linear Regression Models on Cholesterol Efflux Mediated by Plasma in Gilbert Syndrome Patients and Gunn Rats

We further fitted several multiple linear regression models (Table) to evaluate the influence of UCB and blood lipid parameters on the plasma cholesterol efflux capacity in both humans and rats. UCB alone accounted for ≈5% of the variation in efflux within the human samples (*P*<0.01), whereas HDL‐C was the greatest single predictor (8.2%, *P*<0.001). As expected, apo A1 and HDL‐C showed a high level of collinearity (80%, *P*<0.001). The best‐fitting multiple regression model included UCB, HDL‐C, and body mass index (20.7%, *P*<0.001), with HDL‐C remaining the strongest predictor, and UCB contributing to a lesser extent. Age and sex of the participants had no influence on the variability observed in cholesterol efflux.

**Table 1:** Multiple Linear Regression Coefficients for Human and Rat Plasma‐Mediated Cholesterol Efflux

Variable	Human Plasma‐Mediated Cholesterol Efflux		Rat Plasma‐Mediated Cholesterol Efflux	
	Adjusted *R* ^2^	*P* Value	Adjusted *R* ^2^	*P* Value
UCB	0.05	<0.01	0.53	<0.001
HDL‐C	0.082	<0.001	0.78	<0.001
UCB+HDL‐C+BMI	0.207	<0.001	···	···
UCB+HDL‐C+BW+TGC	···	···	0.85	<0.001
HDL‐C+BW+TGC	···	···	0.84	<0.001

The table shows the influence of UCB and HDL‐C as single predictors and the best‐fit regression models for both human and rat on plasma‐mediated cholesterol efflux as a dependent variable. Adjusted *R* ^2^ represents the percentage of explained variation adjusted for the number of observations and variables in the model. BMI, body mass index; BW, body weight; HDL‐C, high‐density lipoprotein cholesterol; TGC, triglycerides; UCB, unconjugated bilirubin.

In the rat cohort, UCB alone accounted for 52% of the variation (*P*<0.001) and HDL‐C for 78% of the variation in the regression model (*P*<0.001). Interestingly, a multiple linear model including both UCB and HDL‐C in rats abolished the influence of UCB in the model, given a high degree of collinearity between HDL‐C and UCB. The best‐fitting multiple regression model contained UCB and HDL‐C in addition to body weight and triglyceride concentrations in rats (85%, *P*<0.001). Removing UCB from this model reduced the adjusted *R* ^2^ value by 0.1%, given its high degree of collinearity with all other variables.

These results indicate that the reduced cholesterol efflux to Gunn rat plasma predominantly depends on HDL‐C concentrations, which appear to be modulated by UCB levels/UGT1A1 function, whereas in humans, additional factors might contribute to plasma‐mediated cholesterol efflux.

### Unconjugated Bilirubin Inhibits Human Plasma‐/Apo A1–Mediated Cholesterol Efflux

**Figure 2:**
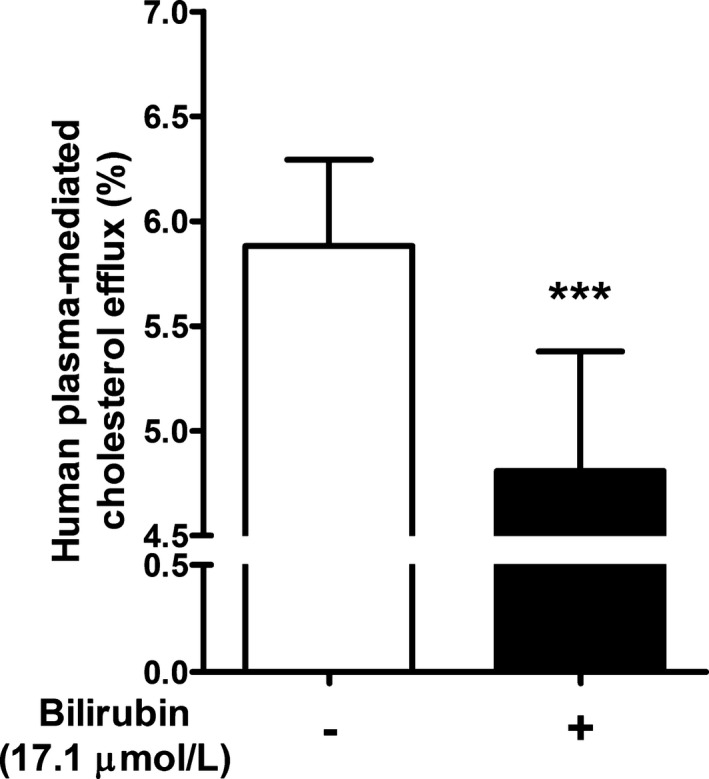
Bilirubin added to normobilirubinemic plasma inhibits cholesterol efflux (n=12). THP‐1 cells were differentiated and loaded as described in Figure 1. Cells were then incubated with fresh serum‐free medium containing human normobilirubinemic plasma (3%, vol/vol) in the absence or presence of bilirubin (17.1 μmol/L) for 4 hours, and cholesterol efflux was determined. Values are mean±SD. ***P<0.001 vs the nontreated group (determined by paired *t* test).

To test whether UCB has an impact on cholesterol efflux, we exogenously supplemented plasma from 12 normobilirubinemic human controls with UCB to a final concentration of 17.1 μmol/L (ie, clinically relevant UCB‐level cutoff for GS diagnosis) and compared it with the naive control plasma of the same participants. The cholesterol efflux mediated by plasma with added UCB was 18.2% lower than that without UCB (*P*<0.0001) (Figure 2).

**Figure 3:**
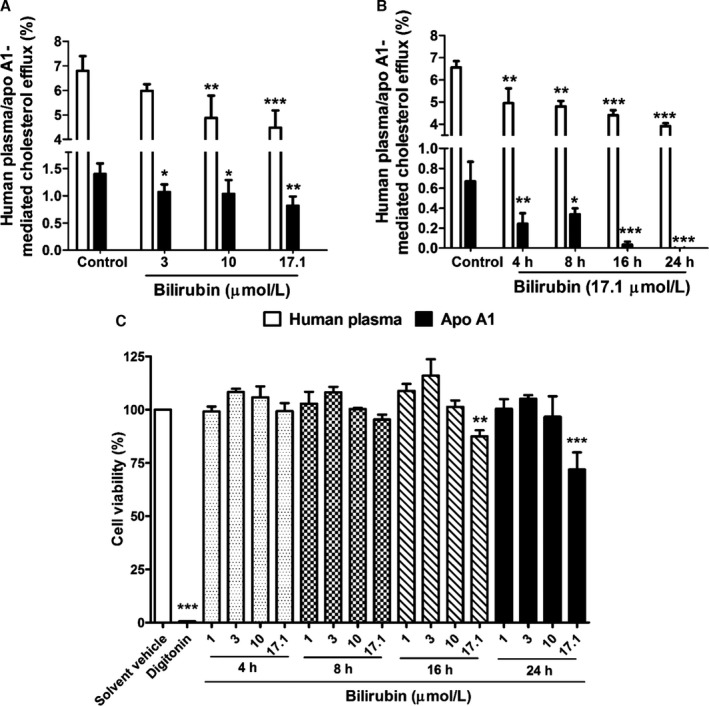
Bilirubin inhibits human plasma‐/apolipoprotein (apo) A1–mediated cholesterol efflux concentration‐ and time‐dependently. A, THP‐1 cells were differentiated and loaded as described in Figure 1. Cells were then incubated with fresh serum‐free medium containing human plasma (3%, vol/vol) or apo A1 (10 μg/mL) in the presence of different concentrations of bilirubin (3, 10, and 17.1 μmol/L) for 4 hours, and cholesterol efflux was determined. Control was treated with solvent vehicle (0.1% dimethyl sulfoxide [DMSO]). B, THP‐1 cells were differentiated and loaded as described in Figure 1. Differentiated cells were incubated with bilirubin (17.1 μmol/L) for 4, 8, 16, and 24 hours. Cells were then incubated with fresh serum‐free medium containing human plasma (3%, vol/vol) or apo A1 (10 μg/mL) for 4 hours, and cholesterol efflux was determined. Control was treated with solvent vehicle (0.1% DMSO). C, Cell viability was determined in the presence of different concentrations of bilirubin for different incubation times. THP‐1 cells were differentiated as described in Figure 1 and then loaded with unlabeled cholesterol for 24 hours. Cells were treated with increasing concentrations of bilirubin (1–17.1 μmol/L) for 4, 8, 16, and 24 hours. The viability was assessed by the resazurin reduction assay. Solvent vehicle treatment (0.1% DMSO) was used as a negative control. The cytotoxic natural product digitonin (50 μg/mL, 4 hours) was used as a positive control. The bar graphs present mean±SD from 3 independent experiments. *P<0.05, **P<0.01, and ***P<0.001 vs control (determined by ANOVA with Bonferroni post hoc test).

**Figure 4:**
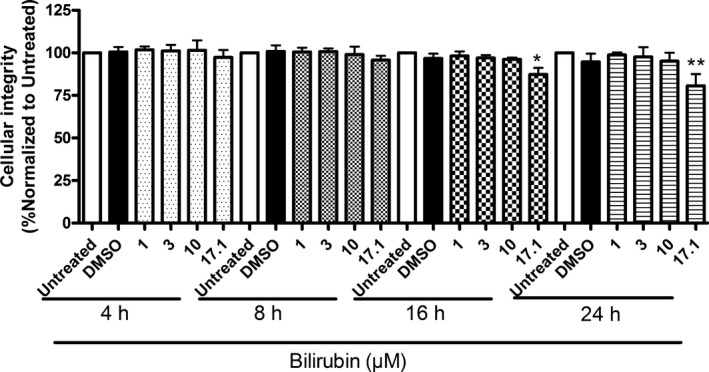
Cellular integrity determined in the presence of different concentrations of bilirubin at different incubation times. THP‐1 cells were differentiated for 72 hours with 200 nmol/L phorbol‐12‐myristate‐13‐acetate and then loaded with unlabeled cholesterol for further 24 hours. Cells were treated with increasing concentrations of bilirubin (1–17.1 μmol/L) for 4, 8, 16, and 24 hours. Cellular integrity was assessed by the Trypan blue exclusion test. Solvent vehicle treatment (0.1% dimethyl sulfoxide [DMSO]) was used as a negative control. The bar graphs present mean±SD from 3 independent experiments. *P<0.05, and **P<0.01 vs solvent vehicle control (determined by ANOVA with Bonferroni post hoc test).

We further observed that UCB inhibited human plasma‐mediated cholesterol efflux in a concentration‐ and time‐dependent manner (Figure 3A and 3B). The inhibition of apo A1–mediated cholesterol efflux was >90% at 16 and 24 hours (Figure 3B), time points when UCB at 17.1 μmol/L slightly reduced cell viability based on a resazurin conversion assay (Figure 3C). Lower concentrations of UCB did not affect cell viability at any time point (Figure 3C). Assessment of cell membrane integrity based on Trypan blue exclusion yielded consistent data (Figure 4).

### Unconjugated Bilirubin Inhibits Cholesterol Efflux by Interacting With THP‐1 Macrophages and Not Cholesterol Acceptors

**Figure 5:**
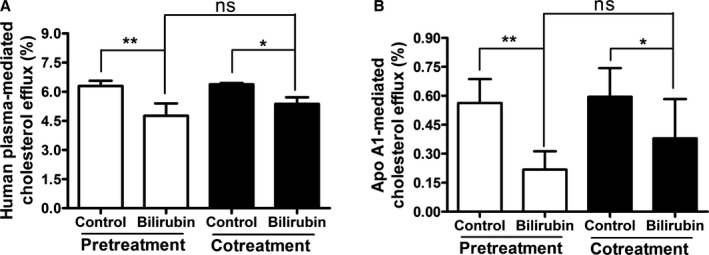
Exposure of THP‐1 macrophages to bilirubin reduces cholesterol efflux, even after bilirubin removal. A, Human plasma as extracellular acceptor and (B) apo A1 as extracellular acceptor. THP‐1 cells were differentiated and loaded as described in Figure 1. For pretreatment, differentiated cells were treated with bilirubin (17.1 μmol/L) for 4 hours and then incubated with fresh serum‐free medium containing human plasma (3%, vol/vol) or apolipoprotein A1 (apo A1; 10 μg/mL) for an additional 4 hours, and cholesterol efflux was determined. For cotreatment, differentiated cells were incubated with fresh serum‐free medium for 4 hours and then incubated with fresh serum‐free medium containing human plasma (3%, vol/vol) or apo A1 (10 μg/mL) in the presence of bilirubin (17.1 μmol/L) for additional 4 hours and cholesterol efflux was determined. Control was treated with solvent vehicle (0.1% dimethyl sulfoxide). The bar graphs present mean±SD from 3 independent experiments. *P<0.05 and **P<0.01 vs control. ns indicates not significant (determined by ANOVA with Bonferroni post hoc test).

To differentiate whether UCB inhibits cholesterol efflux by interfering with cellular (transport) processes in THP‐1 macrophages or by modifying the cholesterol acceptors (human plasma or apo A1), we removed UCB after an incubation time of 4 hours before adding the respective acceptor to THP‐1 macrophages. When macrophages were preincubated with UCB, which was then removed for the efflux experiments, human plasma‐/apo A1–mediated cholesterol efflux remained decreased and was similar to that observed in the presence of both UCB and the cholesterol acceptor (Figure 5). These results suggest that UCB inhibits cholesterol efflux by influencing cellular processes rather than by directly interacting with the used acceptors.

**Figure 6:**
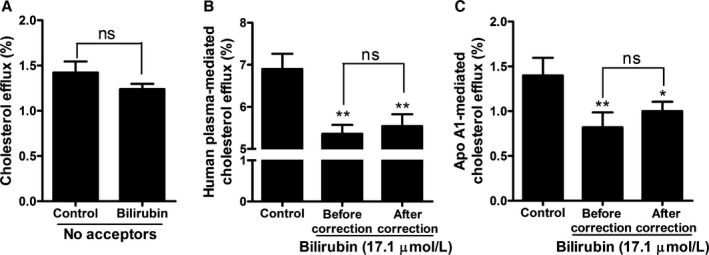
The effect of bilirubin on cholesterol efflux needs human plasma or apolipoprotein A1 (apo A1) as acceptor. A, Bilirubin did not decrease basal cholesterol efflux significantly. THP‐1 cells were differentiated and loaded as described in Figure 1. Differentiated cells were incubated with bilirubin (17.1 μmol/L) for 4 hours in the absence of extracellular acceptors, and cholesterol efflux was determined. Control was treated with solvent vehicle (0.1% dimethyl sulfoxide [DMSO]). B and C, The bilirubin‐induced decrease of cholesterol efflux mediated by (B) human plasma or (C) apo A1 is not different before and after correction for bilirubin's inhibition of basal cholesterol efflux. THP‐1 cells were differentiated and loaded as described in Figure 1. Cells were then incubated with fresh serum‐free medium containing human plasma (3%, vol/vol) or apo A1 (10 μg/mL) in the presence of bilirubin (17.1 μmol/L) for 4 hours, and cholesterol efflux was determined. Control was treated with solvent vehicle (0.1% DMSO). The bilirubin‐induced decrease of cholesterol efflux was corrected for bilirubin's inhibition of basal cholesterol efflux. The bar graphs present mean±SD from 3 independent experiments. *P<0.05 and **P<0.01 vs control. ns indicates not significant (determined by Student *t* test).

We next examined the effect of UCB on basal cholesterol efflux in the absence of any cholesterol acceptor. UCB nonsignificantly decreased the basal cholesterol efflux (Figure 6A). After subtraction of UCB‐induced decrease of the basal cholesterol efflux from the acceptor‐mediated cholesterol efflux, the decrease remained significant (Figure 6B and 6C). Taking into account that acceptor‐mediated efflux involves active transport by membrane transporters and basal efflux is mainly due to passive diffusion, these data suggest that UCB might influence membrane transporters.

### Expression of ABCA1 Protein Is Decreased in THP‐1 Macrophages Treated With Unconjugated Bilirubin and in PBMCs From Gilbert Syndrome Patients

**Figure 7:**
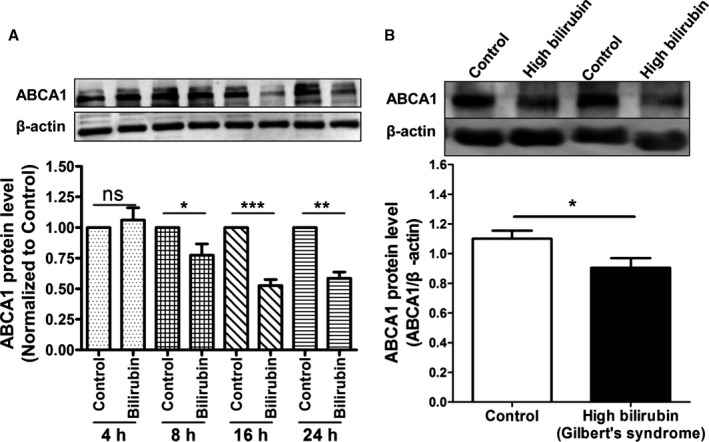
Expression of ATP‐binding cassette transporter A1 (ABCA1) protein in THP‐1 macrophages treated with bilirubin and in peripheral blood mononuclear cells (PBMCs) from Gilbert syndrome (GS) patients. A, Bilirubin suppresses the expression of ABCA1 protein in THP‐1‐derived macrophages time‐dependently. THP‐1 cells were differentiated as described in Figure 1 and then loaded with unlabeled cholesterol for another 24 hours. Cells were treated with bilirubin (17.1 μmol/L) for 4, 8, 16, and 24 hours. The protein levels of ABCA1 were detected by western blotting. Control was treated with solvent vehicle (0.1% dimethyl sulfoxide). The bar graph presents mean±SD from 3 independent experiments. B, Expression of ABCA1 protein is decreased in PBMCs from participants with high bilirubin blood levels (GS) compared with healthy controls. The protein levels of ABCA1 were detected by western blotting. The bar graph presents mean±SEM (n=28 per group). *P<0.05, **P<0.01 and ***P<0.001 vs control. ns indicates not significant (determined by Student *t* test).

**Figure 8:**
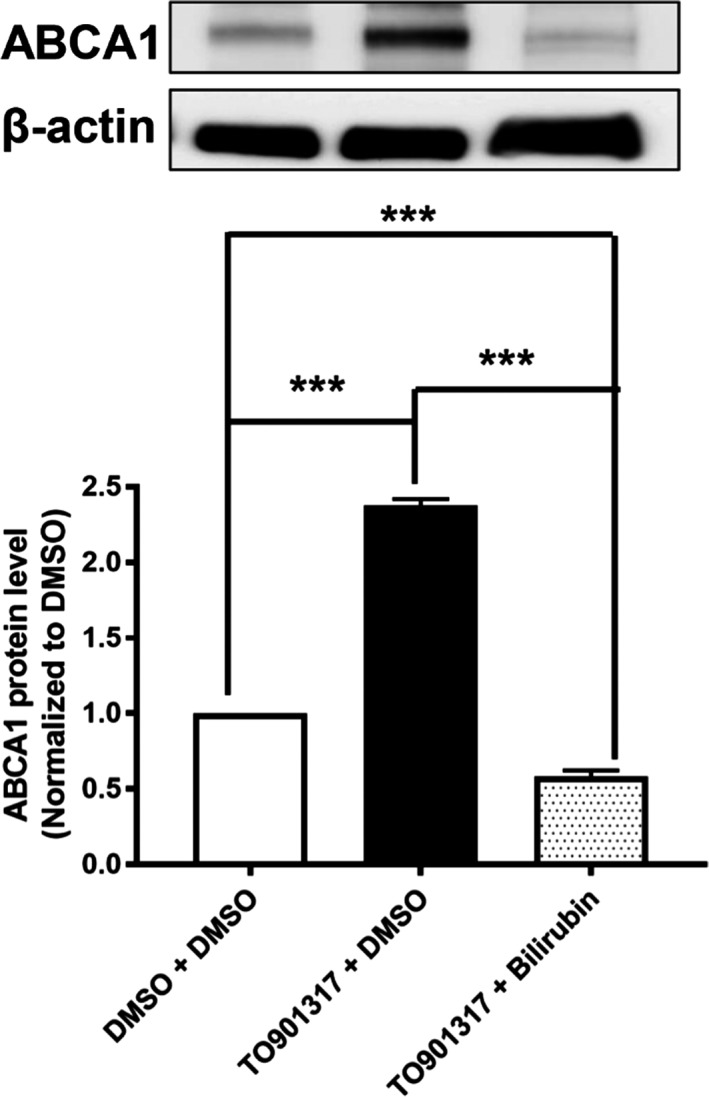
Bilirubin significantly decreases expression of ATP‐binding cassette transporter A1 (ABCA1) protein in THP‐1 macrophages treated with LXR agonist (TO901317) to upregulate ABCA1 protein. THP‐1 cells were differentiated for 72 hours with 200 nmol/L phorbol‐12‐myristate‐13‐acetate and then loaded with unlabeled cholesterol for another 24 hours. Cells were treated with TO901317 (5 μmol/L) or solvent vehicle (0.1% dimethyl sulfoxide [DMSO]) for 24 hours. Afterward, cells were treated with bilirubin (17.1 μmol/L) or solvent vehicle (0.1% DMSO) for 16 hours. The protein levels of ABCA1 were detected by western blot analysis. The bar graphs present mean±SD from 3 independent experiments. ***P<0.001 vs control (determined by ANOVA with Bonferroni post hoc test).

**Figure 9:**
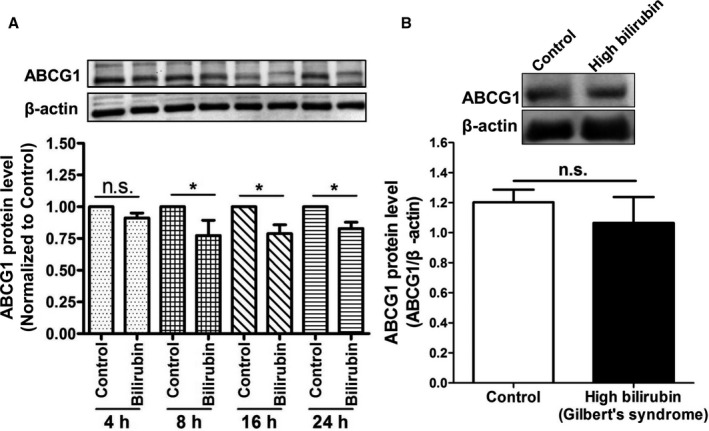
Expression of ATP‐binding cassette transporter G1 (ABCG1) protein in THP‐1 macrophages treated with bilirubin and in peripheral blood mononuclear cells (PBMCs) from Gilbert syndrome (GS) patients. A, Bilirubin suppresses the expression of ABCG1 protein in THP‐1‐derived macrophages. THP‐1 cells were differentiated for 72 hours with 200 nmol/L phorbol‐12‐myristate‐13‐acetate and then loaded with unlabeled cholesterol for another 24 hours. Cells were treated with bilirubin (17.1 μmol/L) for 4, 8, 16, and 24 hours. The protein levels of ABCG1 were detected by western blotting. Control was treated with solvent vehicle (0.1% dimethyl sulfoxide). The bar graphs present mean±SD from 3 independent experiments. B, Expression of ABCG1 protein was not changed significantly in PBMCs from participants with high bilirubin blood levels (GS) compared with healthy controls. The protein levels of ABCG1 were detected by western blotting. The bar graphs present mean±SEM (n=28 per group). *P<0.05 vs control. ns indicates not significant (determined by Student *t* test).

We determined the influence of UCB on protein expression of ABCA1, the major transporter mediating apo A1–induced cholesterol efflux, in THP‐1–derived macrophages at different time points. UCB decreased the expression of the ABCA1 protein in THP‐1–derived macrophages time dependently (Figure 7A), with the first statistically significant reduction evident after 8 hours of incubation. We further explored the protein levels of ABCA1 in PBMCs from human GS patients and controls. ABCA1 protein levels were also significantly decreased in PBMCs from human participants with GS (hyperbilirubinemia) compared with normobilirubinemic control participants (Figure 7B). We also tested an effect of bilirubin on ABCA1 protein expression in THP‐1 macrophages, which were treated with an LXR agonist (T0901317) to strongly induce ABCA1 expression. We observed that bilirubin still downregulates ABCA1 protein levels in the presence of the LXR agonist (Figure 8). In addition to ABCA1, we investigated potential effects on ABCG1 protein levels. Even though a minor reduction of ABCG1 could be observed, the effect was much smaller than the effect on ABCA1 (Figure 9).

### Bilirubin Accelerates the Degradation Rate of the ABCA1 Protein in THP‐1 Macrophages

**Figure 10:**
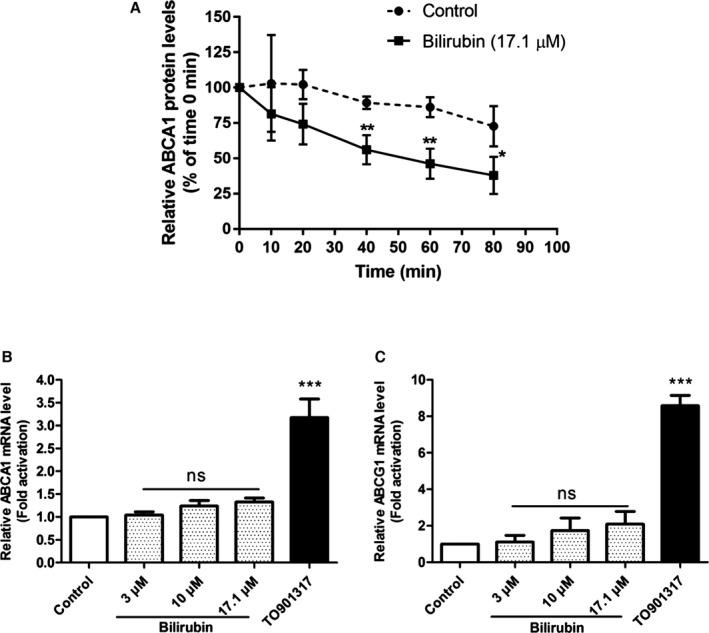
A, Bilirubin enhances the degradation rate of ABCA1 protein in THP‐1 macrophages. THP‐1 cells were differentiated as described in Figure 1 and then loaded with unlabeled cholesterol for another 24 hours. Cells were treated with bilirubin (17.1 μmol/L) for 8 hours. The control was treated with solvent vehicle (0.1% dimethyl sulfoxide) for 8 hours. Cells were lysed after treatment with the protein synthesis inhibitor cycloheximide (140 μmol/L) at different time points (0, 10, 20, 40, 60, and 80 minutes). The protein levels of ATP‐binding cassette transporter A1 (ABCA1) were detected by western blot analysis. The data points present mean±SD from 3 independent experiments. *P<0.05 and **P<0.01 vs control at the same time points (determined by Student *t* test). B and C, Bilirubin does not have a significant effect on mRNA levels of ABCA1 and ATP‐binding cassette transporter G1 (ABCG1) in THP‐1 macrophages. THP‐1 cells were differentiated as described in Figure 1 and then loaded with unlabeled cholesterol for another 24 hours. Cells were treated with bilirubin (3, 10, or 17.1 μmol/L) for 24 hours. The control was treated with solvent vehicle (0.1% dimethyl sulfoxide) for 24 hours. The LXR agonist TO901317 (5 μmol/L) was used as a positive control. The mRNA levels of ABCA1 and ABCG1 were detected by quantitative polymerase chain reaction. Bar graphs present mean±SD from 3 independent experiments. ***P<0.001 vs control (determined by ANOVA with Bonferroni post hoc test). ns indicates not significant.

We next aimed to determine whether the observed changes in ABCA1 protein level induced by UCB were regulated at a protein level by an accelerated degradation rate or at an mRNA level by decreased mRNA expression in THP‐1 macrophages. The effects of bilirubin on ABCA1 protein degradation were tested in the presence of a protein synthesis inhibitor (cycloheximide; 140 μmol/L) by monitoring the decay of ABCA1 protein over time (0, 10, 20, 40, 60, and 80 minutes) in THP‐1 macrophages. As evident from Figure 10A, in the presence of UCB, the degradation rate of ABCA1 protein is significantly higher compared with a nontreated control. Figure 10B and 10C show that neither ABCA1 nor ABCG1 mRNA levels were affected by increased UCB in THP‐1 macrophages.

### ABCA1 Abundance in Livers Is Not Altered in Gunn Rats

**Figure 11:**
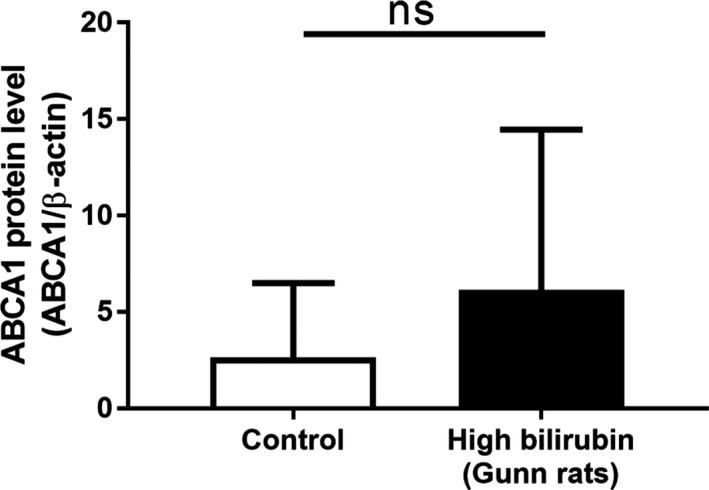
There is no significant difference in expression of ATP‐binding cassette transporter A1 (ABCA1) protein between liver tissues from Gunn rats and Wistar rats. The protein levels of ABCA1 were detected by western blotting. The bar graphs present mean±SD (n=20 per group). ns indicates not significant (determined by Mann–Whitney *U* test). ns indicates not significant.

Previous data from the same cohort show that HDL‐C levels in Gunn rats were significantly lower than that in Wistar rats.^54^ Because the expression level of ABCA1 in the liver has a significant impact on plasma HDL levels^62^ and bilirubin downregulated ABCA1 expression in THP‐1 macrophages, we examined whether the high concentration of serum UCB in Gunn rats decreases ABCA1 protein expression in livers. This would explain lower HDL levels observed in the plasma of Gunn rats compared with Wistar controls. ABCA1 protein levels, however, were not downregulated in Gunn rat liver tissue (Figure 11).

## Discussion

Starting with the hypothesis that an increase of cholesterol efflux capacity induced by bilirubin might be a potential mechanism that contributes to its positive cardiovascular effects, we observed in this work a significant reduction in cholesterol efflux mediated by hyperbilirubinemic plasma obtained from humans and rats. Furthermore, we demonstrated that UCB at physiologically relevant concentrations induces inhibition of cholesterol efflux, which is most likely explained by reduced ABCA1 protein levels. Further study indicates that bilirubin accelerates the degradation of the ABCA1 protein in THP‐1 macrophages. Corroboration of these effects on in vitro addition of UCB and in 2 unique physiological models of hyperbilirubinemia demonstrates the physiological importance and translational relevance of the data obtained.

Recent studies^41^, ^42^, ^43^ indicate that macrophage cholesterol efflux is significantly and inversely associated with CVD, independent of HDL‐C concentration. Macrophage cholesterol efflux, which is the process of macrophage cellular cholesterol export to acceptors in plasma (eg, HDL and apo A1) from cholesterol‐loaded macrophages, plays a key role in the antiatherogenic process of reverse cholesterol transport.^46^, ^63^ Our findings indicate that cholesterol efflux is decreased in the presence of plasma from hyperbilirubinemic participants compared with plasma from normobilirubinemic participants (Figure 1A). Notably, this effect was not restricted to the human species, and a similar pattern was obtained on testing of plasma derived from hyperbilirubinemic rodents (Gunn rats) (Figure 1B). Although in humans both groups exhibited similar profiles of lipoprotein cholesterol acceptors such as HDL‐C and apo A1, multiple regression models indicated that HDL‐C was among the best predictors of the observed effect but also indicated a possible independent effect of UCB concentrations. Exogenously added UCB at a concentration of 17.1 μmol/L (ie, clinically relevant UCB‐level cutoff for GS diagnosis) suppressed cholesterol efflux (Figure 2). Furthermore, UCB added in vitro at a final concentration of 3 μmol/L, which is on the lower end of the physiological concentration of UCB in healthy persons, could already show a significant reduction of cholesterol efflux.

Reduced cholesterol efflux from macrophages was observed as early as 4 hours after incubation with UCB. This indicates that decreased cholesterol efflux is a direct result of UCB exposure. Moreover, the effect appeared more pronounced with prolonged exposure as cholesterol efflux continued to decline until 24 hours of incubation. Although cells showed a moderate decline in viability after 16 and 24 hours of incubation with 17.1 μmol/L UCB, likely due to accumulation of potentially cytotoxic bilirubin or its oxidation products,^64^ no cytotoxicity was observed with UCB concentrations <10 μmol/L, even after prolonged exposure.

Preincubation of macrophages with UCB also reduced cholesterol efflux compared with control plasma. We thus conclude that UCB induces cellular changes that account for the observed effects (Figure 5) rather than interfering with acceptor proteins, as published previously.^65^ Lack of activity within basal efflux experiments suggested that the effect of UCB is induced by influencing transporter‐mediated cholesterol efflux (Figure 6).

In macrophages, ABCA1 is a key plasma membrane protein required for the efflux of cellular cholesterol to extracellular acceptors and is confirmed as a key mediator of macrophage cholesterol efflux to HDL.^59^, ^66^ Deletion of ABCA1 in macrophages enhances atherosclerosis in mice.^67^ In humans, mutations in ABCA1 cause Tangier disease, a severe HDL deficiency syndrome characterized by accumulation of cholesterol in tissue macrophages and prevalent atherosclerosis.^68^ Conversely, overexpression of ABCA1 in macrophages reduced atherosclerosis.^69^ ABCA1 is the major transporter mediating cholesterol efflux to apo A1, and the fact that UCB exhibited very similar pattern of action in the presence of plasma and apo A1 (Figure 3) indicated that this transporter is a likely target of UCB action. Indeed, western blotting revealed that UCB reduced ABCA1 protein levels in THP‐1 macrophages (Figure 7). In addition, participants with GS had lower ABCA1 protein levels in PBMCs compared with control participants. It is unclear whether this decrease in ABCA1 expression in vivo reflects a transient effect caused by an acute increase in UCB in GS patients due to a presampling caloric restriction^52^, ^53^ or if GS patients have constitutively reduced ABCA1 protein levels. The mechanism by which UCB downregulates ABCA1 protein levels is accelerated degradation of ABCA1 rather than decreased synthesis. We also tested whether bilirubin could repress ABCA1 protein expression under conditions in which the cells were treated with an LXR agonist (T0901317) to strongly induce ABCA1 expression. We observed that bilirubin still downregulates ABCA1 protein levels even in the presence of the LXR agonist (Figure 8).

In this study, we also examined ABCA1 protein expression in livers of Gunn rats and respective Wistar controls, which could potentially explain the observed lower HDL levels in the plasma of Gunn rats compared with Wistar controls. Contrary to our expectations, high concentrations of serum UCB in Gunn rats were not associated with lower ABCA1 protein levels in livers. Several hypotheses exist to explain how bilirubin might lead to a decrease of HDL‐C in Gunn rats. A contributor to HDL formation is cholesterol efflux from macrophages mediated by ABCA1.^70^ Our results suggest that the reduction of macrophage cholesterol efflux by bilirubin could possibly contribute, albeit to a low extent, to the observed reduction of HDL‐C in Gunn rats. In this context, more research is needed to investigate the role of bilirubin on ABCA1 expression and cholesterol efflux capacity of other tissues and organs (eg, the intestine) that have a more pronounced effect on HDL‐C levels in plasma. Recent studies also suggest that bilirubin could influence lipid metabolism and reduce total cholesterol in serum.^71^, ^72^ Possible effects of bilirubin on cholesterol biosynthesis, which might contribute to reduced total cholesterol—including LDL‐C and HDL‐C in Gunn rats^2^—remain to be studied.

It is essential to note that although both Gunn rats and GS patients represent 2 independent models of unconjugated hyperbilirubinemia, several important differences exist in their ability to metabolize and transport cholesterol and bilirubin. Gunn rats have almost no UGT1A1 activity compared with ≈30% remaining conjugation activity in human GS patients.^2^, ^8^, ^12^ As a result, Gunn rats usually possess a 50‐fold increase in UCB concentration compared with wild‐type controls, whereas GS patients typically demonstrate 3‐ to 4‐fold increase in UCB concentration compared with persons without GS.^2^, ^8^ Another general difference, is that HDL constitutes the greatest lipoprotein fraction in rats, as opposed to humans, whose HDL concentrations typically constitute ≈25% of total circulating cholesterol.^2^

Our results may contribute to a better understanding of the complex pattern of action of bilirubin in the context of CVD, including the previously observed U‐shaped relationship between circulating bilirubin concentrations and risk of CVD.^23^, ^24^, ^25^, ^26^, ^27^, ^28^, ^29^, ^30^ Given the multitude of factors contributing to CVD, it is plausible that bilirubin affects various relevant cellular processes. Bilirubin has very strong antioxidant activity, which is almost 30 times more potent in preventing LDL oxidation than a vitamin E analogue.^73^ High‐normal plasma UCB levels (10–17.1 μmol/L) might prevent LDL oxidation, reducing CVD risk, whereas higher concentrations of UCB might suppress ABCA1 levels and promote foam cell formation.

In summary, we showed for the first time that macrophage cholesterol efflux is significantly impaired in the presence of plasma from GS patients and Gunn rats with hyperbilirubinemia. Furthermore, we demonstrate that UCB inhibits macrophage cholesterol efflux and suppresses the protein levels of ABCA1. Further study indicates that bilirubin increases the degradation rate of the ABCA1 protein in THP‐1 macrophages. These novel data underscore the complex bioactivity of bilirubin in the context of CVD and may encourage further exploration of bilirubin's effect on cholesterol metabolism and transport.

## Sources of Funding

This work was supported by grants from the Austrian Science Fund (FWF): P25971‐B23 and the Vienna Anniversary Foundation for Higher Education (Hochschuljubiläumsstiftung der Stadt Wien): H‐297332/2014, as well as by the University of Vienna (Research Platform Active Ageing). Wang was supported by a Chinese Government Scholarship from the China Scholarship Council (CSC No. 201406240043).

## Disclosures

None.
